# Pro-Inflammatory Action of MIF in Acute Myocardial Infarction via Activation of Peripheral Blood Mononuclear Cells

**DOI:** 10.1371/journal.pone.0076206

**Published:** 2013-10-01

**Authors:** David A. White, Lu Fang, William Chan, Eric F. Morand, Helen Kiriazis, Stephen J. Duffy, Andrew J. Taylor, Anthony M. Dart, Xiao-Jun Du, Xiao-Ming Gao

**Affiliations:** 1 Baker IDI Heart and Diabetes Institute, Melbourne, Australia; 2 Department of Cardiovascular Medicine, Alfred Hospital, Melbourne, Australia; 3 Centre for Inflammatory Diseases, Monash University, Melbourne, Australia; 4 Department of Medicine, Central Clinical School, Monash University, Melbourne, Australia; 5 Department of Surgery, Central Clinical School, Monash University, Melbourne, Australia; King’s College London School of Medicine, United Kingdom

## Abstract

**Objectives:**

Macrophage migration inhibitory factor (MIF), a pro-inflammatory cytokine, has been implicated in the pathogenesis of multiple inflammatory disorders. We determined changes in circulating MIF levels, explored the cellular source of MIF, and studied the role of MIF in mediating inflammatory responses following acute myocardial infarction (MI).

**Methods and Results:**

We recruited 15 patients with MI, 10 patients with stable angina and 10 healthy volunteers and measured temporal changes of MIF in plasma. Expression of MIF, matrix metalloproteinase-9 (MMP-9) and interleukin-6 (IL-6) in cultured peripheral blood mononuclear cells (PBMCs) and the media were measured by ELISA or real-time PCR. Compared to controls, plasma levels of MIF and IL-6 were significantly elevated at admission and 72 h post-MI. In contrast, expression of MIF, MMP-9 and IL-6 by PBMCs from MI patients was unchanged at admission, but significantly increased at 72 h. Addition of MIF activated cultured PBMCs by upregulating expression of inflammatory molecules and also synergistically enhanced stimulatory action of IL-1β which were inhibited by anti-MIF interventions. In a mouse MI model we observed similar changes in circulating MIF as seen in patients, with reciprocal significant increases in plasma MIF and reduction of MIF content in the infarct myocardium at 3 h after MI. MIF content in the infarct myocardium was restored at 72 h post-MI and was associated with robust macrophage infiltration. Further, anti-MIF intervention significantly reduced inflammatory cell infiltration and expression of monocyte chemoattractant protein-1 at 24 h and incidence of cardiac rupture in mice post-MI.

**Conclusion:**

MI leads to a rapid release of MIF from the myocardium into circulation. Subsequently MIF facilitates PBMC production of pro-inflammatory mediators and myocardial inflammatory infiltration. Attenuation of these events, and post-MI cardiac rupture, by anti-MIF interventions suggests that MIF could be a potential therapeutic target following MI.

## Introduction

Acute myocardial infarction (MI) triggers regional and systemic inflammatory responses. Ischemic and necrotic cardiomyocytes release a range of pro-inflammatory cytokines and chemokines that recruit inflammatory cells to the ischemic area, facilitating the wound healing process. However, excessive regional inflammatory responses may amplify tissue damage by promoting cardiomyocyte death [1]. Further, excessive production and activation of matrix metalloproteinases (MMPs), particularly MMP-9, causes degradation of collagen matrix resulting in cardiac remodeling and subsequent dysfunction [1,2]. Indeed, we have documented in the mouse that post-MI cardiac rupture, an extreme form of acute cardiac remodeling, is a consequence of severe inflammation and extracellular matrix damage [3]. The degree of inflammatory responses following MI is an important determinant of clinical outcomes [4]. In patients with acute MI, higher white blood cell and monocyte count at admission are associated with poorer prognosis [5,6]. Although a number of animal studies have shown beneficial effects of anti-inflammatory strategies in reducing infarct size or attenuating cardiac remodelling [7,8], clinical trials testing anti-inflammatory therapies have generally proven disappointing [9,10]. Therefore, successful modulation of acute inflammatory responses following MI requires more precise understanding of the mechanisms involved.

Macrophage migration inhibitory factor (MIF), a pleiotropic cytokine, is believed to control the inflammatory ‘set point’ by regulating the release of other pro-inflammatory mediators [11]. MIF has been implicated in the pathogenesis of a wide range of inflammatory disorders such as septic shock, diabetes, colitis, rheumatoid arthritis and glomerulonephritis [11–13]. Recent studies have indicated that MIF promotes progression of atherosclerosis and plaque instability [14–16]. Increased expression of myocardial MIF has been observed in a rat model of MI [17]. Elevated plasma levels of MIF were also reported in patients with MI [18,19], but not in those with unstable angina, suggesting that MIF may be released from necrotic cardiomyocytes. Our previous study demonstrated that activation of peripheral blood mononuclear cells (PBMCs) in MI patients was associated with upregulation of an array of inflammatory genes, implying significant roles for PBMCs in systemic and regional inflammatory responses and ECM remodelling in MI [20]. However, the cellular source of elevated circulating MIF and the potential significance of MIF in promoting inflammation and related consequences are not known.

Thus, in this study, we examined plasma MIF levels at different time-points after MI in human patients. Using a mouse MI model and cultured human PBMCs, we investigated dynamic changes of circulating MIF and the role of MIF in promoting inflammatory responses.

## Materials and Methods

### Studies in Patients with MI

#### Study Participants

Consecutive patients who had their first MI presenting to the Alfred Hospital that satisfied the following criteria were recruited: (1) typical and persistent chest pain; (2) electrocardiographic (ECG) signs of ST-segment elevation ≥ 2 mm and/or pathological Q waves in ≥ 2 consecutive pre-cordial leads or ≥ 1 mm in limb leads; and (3) a typical rise and fall of the cardiac biomarker, troponin-I. Routine laboratory tests for serial cardiac troponin-I and creatine kinase were conducted and all patients underwent primary percutaneous coronary intervention (PCI). Aspirin, clopidogrel, nitrate and heparin were administered in all cases after admission and/or during PCI as per standard clinical practice and the majority received angiotensin-converting enzyme (ACE) inhibitors, statin and β-blockers following PCI. Patients with active infection or chronic inflammatory diseases or who were receiving anti-inflammatory medications were excluded. However, it should be aware a potential influence by other co-morbidities such as hypertension, diabetes and hyperlipidemia.

Ten healthy volunteers (no signs of coronary artery disease) with ages similar to the acute MI patients were recruited as controls. Another 10 patients with stable angina (e.g. effort-induced minutes chest discomfort/pain) without typical ECG and enzymatic changes or MI in the past 12 months were also recruited as non-infarct controls during routine outpatient visits. This study complied with the Declaration of Helsinki and was approved by the Ethics Committee of the Alfred Hospital. Informed written consent was obtained from all participants.

#### Blood Sampling

Blood samples were collected using heparinised and EDTA vacuum tubes from healthy volunteers or from patients with stable angina. For patients with MI, the first blood sample was collected at the time of hospital admission (average 3 h after onset of symptoms) prior to PCI and the second samples at 72 h after MI. Blood samples were processed within 30 min after collection, heparinised plasma was separated by centrifuge at 3000 g for 15 min at room temperature and stored at -80°C until assay.

#### Routine Laboratory Tests

White blood cell count at admission was performed by Alfred Pathology.

#### Isolation of PBMCs

PBMCs from MI patients and control volunteers were isolated from whole blood collected in EDTA tubes using Ficoll-Paque plus (Amersham Biosciences) according to the manufacturer’s instructions. Briefly, whole blood was layered on to the top of Ficoll-Paque and centrifuged at 400*g* for 30 min at room temperature. The mononuclear cell layer was carefully collected and rinsed twice with PBS, as previously described [20].

#### Proteins, Antibodies and Chemicals used in cell culture

**Figure 1:**
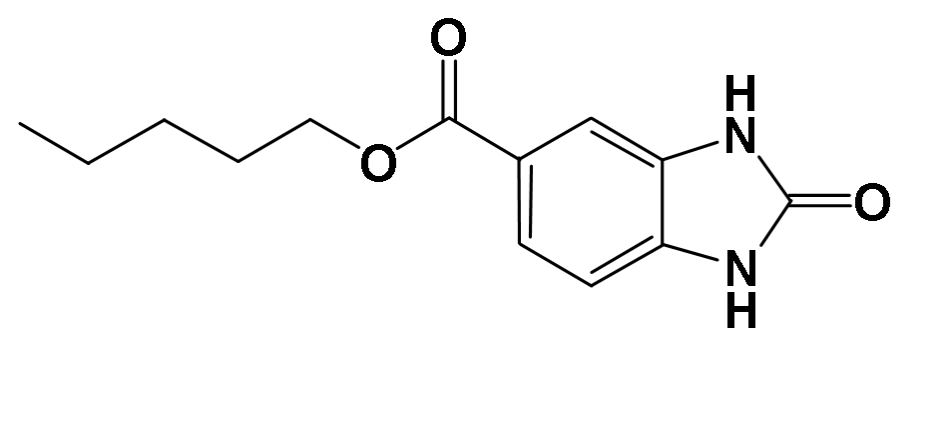
Molecular structure of small molecular MIF antagonist, COR100140. Molecular weight 248.28.

Recombinant human MIF (rMIF) and IL-1β were purchased from eBioscience™ (San Diego CA, USA) and Millipore (Temecula, CA, USA), respectively. Two anti-MIF agents were studied. COR100140 is a small molecule MIF antagonist with molecular weight 248.28 (donated by Cortical Pty Ltd) which interacts with the MIF tautomerase catalytic site and prevents its bioactivity [16]. The molecular structure of COR100140 is shown in Figure 1. Anti-MIF monoclonal antibody was academic supply from Dr Jie Tang (Institute of Biophysics of Chinese Academy of Sciences) to neutralize MIF bioactivity [21].

#### Protocol of Cell Culture Experiments

After isolation, PBMCs were cultured with RPMI 1640 medium (Sigma) supplemented with L-glutamine and antibiotics/antimyocyocotic at a density of 2.5×10^6^ cells/ml and incubated at 37°C for 24 h. PBMCs from MI patients were cultured either with vehicle medium (control, with or without DMSO), COR100140 (50 μM, dissolved in DMSO), anti-MIF monoclonal antibody (10 µg/ml) or isotype control antibody (10 µg/ml, LEAF^TM^ mouse IgG2b, Biolegend). PBMCs prepared from control volunteers were also cultured with (1) recombinant human MIF (rMIF, 5 ng/ml, eBioscience), and/or interleukin-1β (IL-1β, 10 ng/ml); (2) with IL-1β (10 ng/ml) and COR100140 (50 μM) or anti-MIF monoclonal antibody (10 μg/ml). In our pilot experiment, we tested the dose-effect of COR100140 on IL-1β-induced activation of PBMCs determined by gelatine zymography for MMP-9 expression, and observed that COR100140 at 50 μM completely attenuated IL-1β induced MMP-9 expression (Figure S1). The concentration of anti-MIF monoclonal antibody (10 µg/ml) used was shown to significantly inhibit the stimulatory effect of tumor necrotic factor α (TNFα) in myoblasts [22]. The rMIF concentration (5 ng/ml) is similar to the peak plasma level of MIF in patients at day 1 after MI [19]. The IL-1β concentration of 10 ng/ml has been shown to induce maximal MIF production in cultured human cells [23]. After 24 h culture, PBMCs and culture media were harvested for the following assays (described below). This cell culture protocol was established by our previous study [20]. Since there was no difference between vehicle medium (with or without DMSO) and isotype control antibody IgG2b on expression of MIF, MMP-9 and IL-6 at mRNA and protein levels, we combined the results from these controls.

#### Enzyme-Linked Immunosorbent Assay (ELISA)

Levels of MIF, MMP-9, IL-6 and IL-1β in plasma or cell culture media were assessed using DuoSet ELISA Kits from R&D Systems (DY289 for MIF; DY911 for MMP-9; DY206 for IL-6 and DY201 for IL-1β) according to the manufacturer’s instructions.

#### Gene Expression

Total RNA was extracted from PBMCs following 24 h incubation. After reverse transcription, quantitative real-time PCR was performed using a SYBR green mix (Invitrogen) and on the ABI Prism 7500 system (Applied Biosystems) to measure MIF, MMP-9, IL-6 and IL-1β. Expression levels were calculated using the method of 2-ΔΔct and normalized to housekeeping gene GAPDH, as previously described [3].

### Murine MI model

To identify the source of MIF that was released into the circulation during the acute phase of MI, we used a mouse MI model to determine simultaneously the changes of MIF content in plasma and the infarct myocardium. Effect of anti-MIF interventions on the inflammatory response and cardiac remodelling were also studied.

#### Induction of MI and Anti-MIF Intervention

Male C57Bl/6 mice at 10 weeks of age were used. All procedures were approved by AMREP Animal Ethic Committee in accordance with the Australian Code of Practice for the Care and Use of Animals for Scientific Purposes. After anaesthesia with a mixture of ketamine, xylazine and atropine (100, 20 and 1.2 mg/kg, respectively, i.p.), mechanically ventilated mice were subjected to coronary artery occlusion (CAO) for 3 h, 24 h, 72 h, 7 days or 4 weeks, respectively, or sham operation [3]. At the end of the planned ischemia period, blood was collected by cardiac puncture and plasma separated and stored at -80°C for further assay. The heart was collected and either fresh frozen and embedded in O.C.T. for immunohistochemistry or formalin fixed and embedded in paraffin for histological study.

To investigate the effect of anti-MIF intervention on inflammatory responses, animals were treated with a single dose of anti-MIF polyclonal antibody (Santa Cruz, sc-20121) or isotype control rabbit IgG (Santa Cruz, sc-2027), at 5 mg/kg i.p. immediately after CAO. Hearts were collected at 24 h and 7 days for study of inflammatory cell infiltration. Further, to investigate the influence of MIF inhibition on cardiac remodeling and function, another batch of mice were treated with COR100140 at a daily dose of 50 mg/kg by gavage for the first 3 days after CAO; this timing was designed to avoid an induction of cardiac rupture due to handling stress. Animals were studied for 4 weeks by echocardiography. Autopsy was performed on all mice found dead to identify the cause of death. In all experiments, animals were closely monitored according to a standard procedure approved by the Animal Ethic Committee.

#### Echocardiography

Echocardiography was performed in mice at baseline, 2 and 4 weeks after CAO, time-points selected based on the documented process of post-MI myocardial healing and remodeling in mice [24]. Briefly, mice were anesthetised with isoflurane at a flow rate of 1.7%. Using an iE33 ultrasound system and a 15MHz linear probe (Philips), a short-axis view of the left ventricle (LV) close to papillary muscles was obtained. A 2 D guided M-mode trace crossing the anterior and posterior wall of the LV was acquired. Images were analysed by a single investigator in a blinded fashion. LV dimensions at end-diastole and end-systole (LVEDd, LVESd), posterior wall thickness at diastole and systole (PWd th, PWs th) were measured and fractional shortening (FS%) was calculated [24].

#### Immunohistochemistry

Temporal changes of the density of macrophages in the infarct myocardium and influence of anti-MIF antibody treatment on macrophage infiltration following MI were examined by immunohistochemistry. Briefly, fresh-frozen LV sections were incubated with rat anti-mouse CD45 antibody for leukocytes (1:50, BD Pharmingen) or CD68 antibody for macrophages (1:200, Serotec) for 1 h followed by incubation with secondary antibody Alexa Fluor 546 goat anti-rat IgG (1:1000, Invitrogen) for 30 min. Nuclei were stained by nuclear acid dye, 4', 6-diamidino-2-phenylindole (DAPI) (Invitrogen, 1:1000). Overlayed images of CD45 or CD68 positive stained cells with DAPI stained nucleus were identified as positive staining. Multiple images (8-10 per heart) covering the entire infarct region of the LV section were acquired digitally using Olympus BX61 fluorescence microscope and AnalySIS FIVE software (Olympus) at ×20 magnification and the number of inflammatory cells were counted manually in a blinded fashion, as previously described [25,26].

#### Immunoblotting

Proteins were extracted from LV tissues and separated on a 10% SDS-PAGE before transfer to a PVDF membrane. The membrane was blocked with 5% skim milk in TBST and then incubated with primary antibodies, for CD74 (1:1000, Santa Cruz Technologies Biotechnologies, TX, USA) and monocyte chemoattractant protein-1 (MCP-1, 1:2000, Abcam, NSW, Aust.), overnight at 4°C followed by incubation with corresponding secondary antibody conjugated with horseradish peroxidise (Santa Cruz Technologies Biotechnologies). Proteins were visualised by enhanced chemiluminescence reagent (Millipore, USA) and quantified using Quantity One software (Version 4.5.2, Bio-Rad Laboratories, USA), as reported previously [25]. Membranes were re-probed with α-tubulin antibody (Sigma-Aldrich, Aust.) to verify loading consistency.

#### Histology

As the inflammatory response is necessary for wound healing, to understand whether MIF has an influence on healing, we measured healing-related parameters in the LV sections from mice with or without anti-MIF polyclonal antibody treatment at 7 days post MI. Hearts were stained with hemotoxylin and eosin (H&E) and digital images of LV sections were obtained at ×4 magnification to cover the whole infarct segment, and edges of the infarct region and residual coagulative necrotic myocardium within the infarct region were traced and the area was calculated. Necrotic area was expressed as percentage of the entire infarct region. Infarct wall thickness (at ×10 magnification, averaged from 5 measurements) was determined from H&E stained LV sections. For collagen quantification, 8-10 images (at ×10 magnification) were obtained from Sirius red stained LV sections, percentage of collagen content (red stained area) in the infarct region was measured [27]. Infarct size was determined as percentage of infarct segment length in the length of entire LV segment from serially H&E stained LV sections (at ×4 magnification), as described previously [28]. Image-Pro Plus 6.0 software (Media Cybernetics, Inc, USA) were used for all image analyses.

#### MIF ELISA

MIF concentrations in mouse plasma and the infarct myocardium were measured in duplicate by ELISA using a commercially available mouse MIF kit (EIAab Science Co. Ltd, Wuhan, China) according to the manufacturer’s instructions.

#### Statistical Analysis

Results are presented as mean±SEM, unless otherwise stated. Graphpad Prism 5.0 was used for statistical analyses. Unpaired Student *t* test, one-way or two-way ANOVA was used where appropriate to detect significance between groups with Tukey multiple comparison for *post hoc* test. Rupture incidence was analysed by Fisher exact test. *P<0.05* was considered statistically significant.

## Results

### Clinical Characteristics

Of the patients with MI (n=15) and stable angina (n=10), the majority were males. Patients with stable angina tended to be older. More patients with MI were smokers while there were no significant differences in other known risk factors, such as frequency in diabetes, hypertension, hyperlipidemia or family history of coronary artery among the 3 groups (Table 1). In MI patients, locations of the infarct were 7 in anterior, 6 in inferior and 2 in posterior wall. Clinical characteristics of patients with MI are summarized in Table 1.

**Table 1:** Clinical characteristics of study participants.

	Healthy control	Stable angina	Acute MI	P value CTL vs. MI
n	10	10	15	-
Age (years)	59±13	72±10*	62±10†	0.52
Gender (n, male/female)	5/5	8/2	10/5	0.44
Body surface area (m^2^)	1.8±0.16	1.98±0.15	1.95±0.16*	0.03
Smoking (%)	10	20	53*†	0.04
Hypertension (%)	20	40	27	1.00
Diabetes (%)	20	30	20	1.00
Hyperlipidemia (%)	40	40	27	0.67
Family History of CAD (%)	40	50	46	1.00
WBC (10^3^/μL)	6.2±1.5	7.4±1.7	9.9±2.5*†	0.0004
Monocytes (10^3^/μL)	0.5±0.2	0.6±0.2	0.9±0.3	0. 01
Peak troponin I (μg/L)	-	-	50.2±42.8	
Peak creatine kinase (units/L)	-	-	1530±995	
Number of stenosed vessels	-	-	1.7±0.66	
Time from symptom to reperfusion (min)	-	-	188±67	

Values are expressed as mean±SD, percentage or exact number. CAD, coronary artery disease in immediate family. WBC, white blood cell; MI, myocardial infarction. CTL, healthy control. *P<0.05 vs. CTL, † *P*<0.05 vs. stable angina.

### Rapid Rise in Circulating Level of MIF and Evidence for the Cardiac Origin following MI

**Figure 2:**
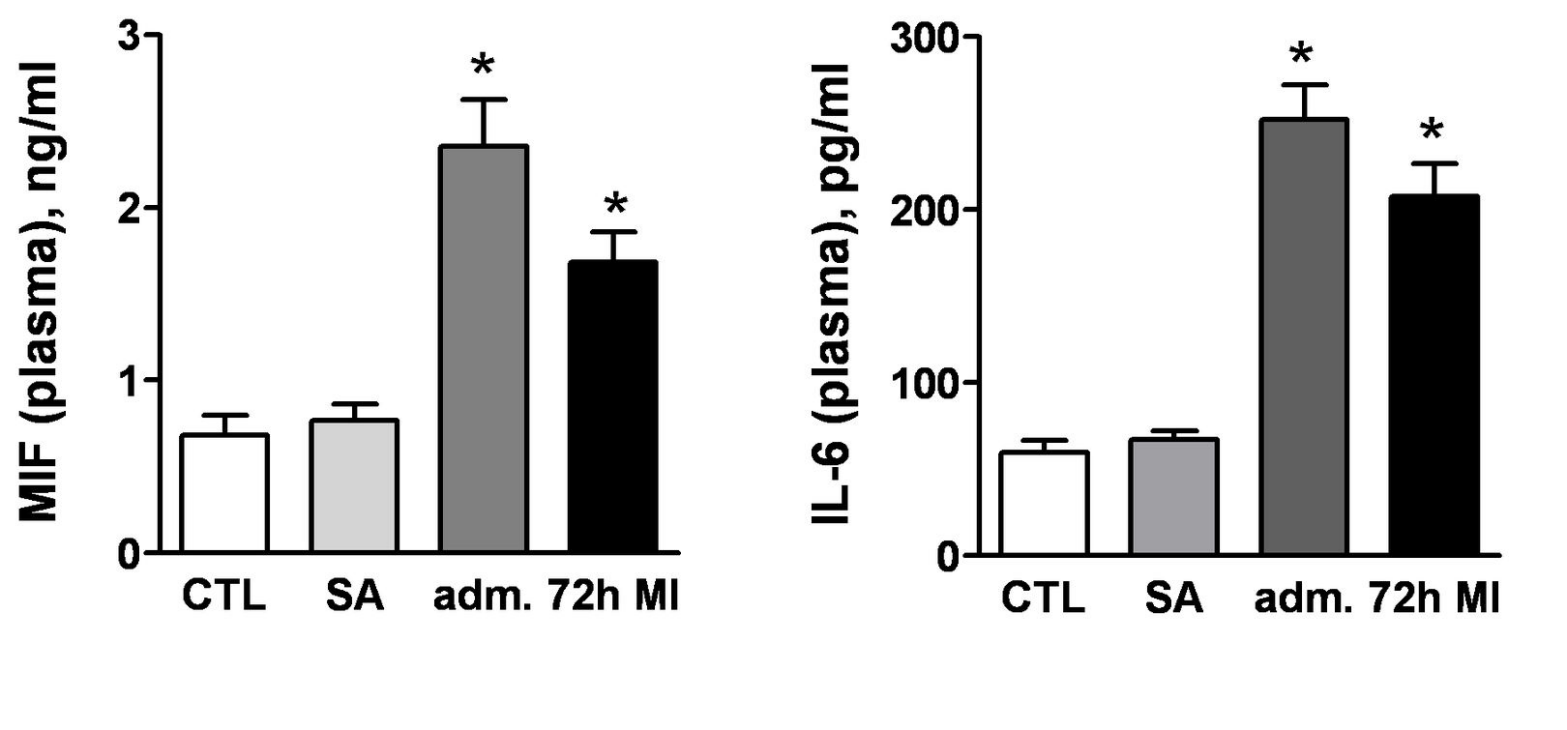
Significant increase in plasma levels of MIF and interleukin-6 (IL-6) in patients at admission (average 3 h after onset of symptoms) and 72 h following MI. n=15 for patients with MI, n=10 for stable angina (SA) and n=10 for healthy controls (CTL). *P<0.05 vs. CTL or SA.

At admission (average 3 h after onset of symptoms), plasma level of MIF was 3.2-fold higher than the two control groups, healthy control and stable angina (Figure 2). This elevation was sustained at 72 h after MI although a decline was observed (Figure 2). In addition, plasma IL-6 levels also had a similar temporal change. The elevated admission plasma MIF was associated with early rise of IL-6, and increased white blood cell and monocyte counts at admission (Table 1).

In regards to the variation of human plasma MIF values in published studies, except different ELISA kits applied, a recent study has reported that a delayed processing, hemolysis and anticoagulant used for blood collection (elevated MIF value in EDTA) would increase MIF values significantly [29]. In the current study we followed the sample processing protocol described in Method part and obtained stable and reliable MIF results.

**Figure 3:**
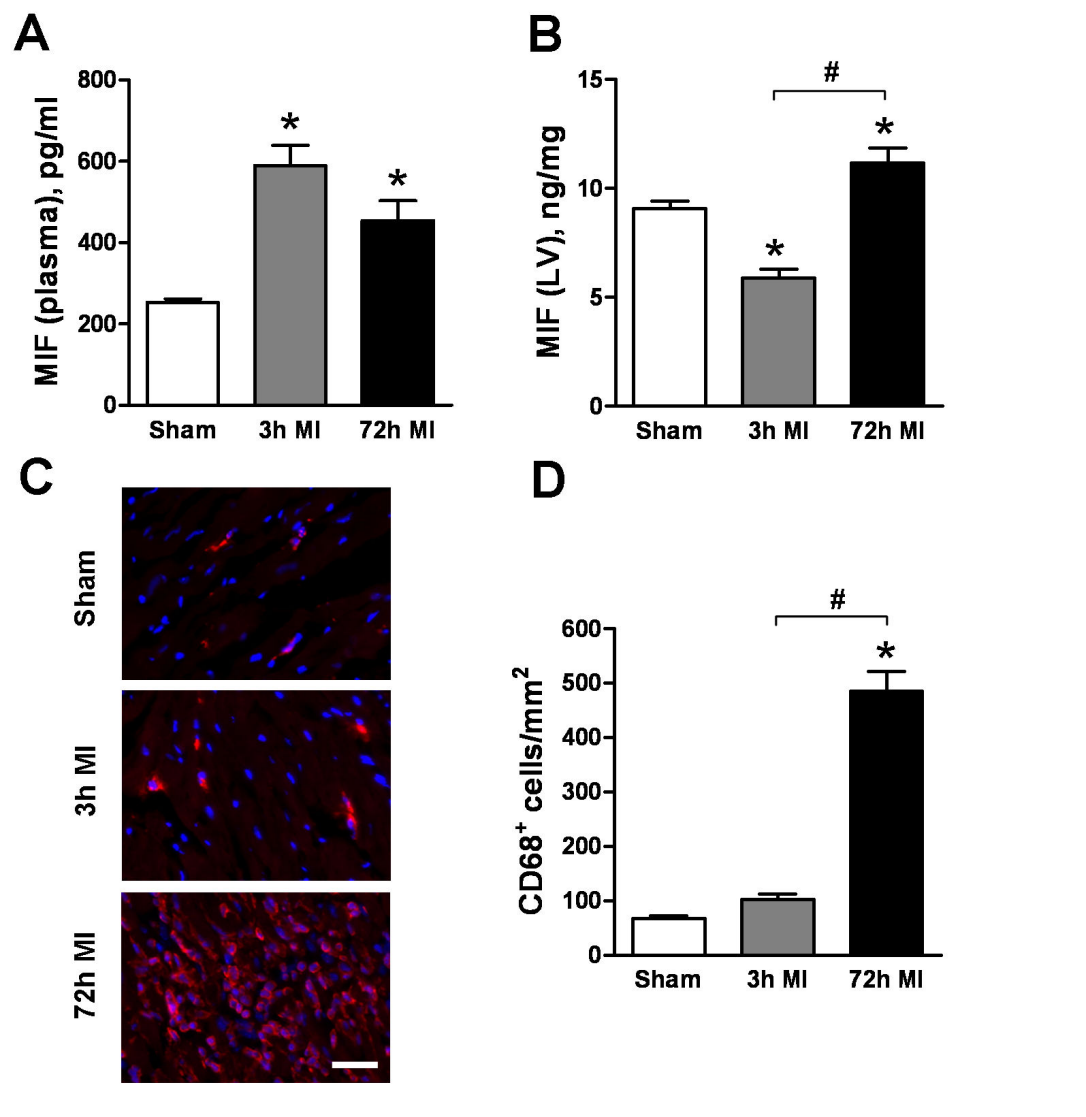
Changes of MIF levels in plasma and the myocardium and inflammatory cells infiltration in a mouse MI model. Temporal changes of MIF levels in plasma (**A**) and the infarct myocardium (**B**) following coronary artery occlusion. Notably, a reciprocal change of MIF content in plasma and the infarct myocardium occurred at 3 h post MI. **C**. representative images to elucidate time-dependent increase in the density of CD68 positive-stained macrophages in the infarct myocardium, bar=100 μm. n=5-8/group. *P<0.05 vs. sham. ^#^ *P<0*.*05*.

To define the source of the early rise of circulating MIF, we studied mice subjected to MI and measured MIF content in plasma and the infarct myocardium at time points matched with those observed in patients. A significant elevation of plasma MIF level was detected in mice with MI at 3 h which persisted up to 72 h (Figure 3A), which were similar to that seen in MI patients. Notably, after MI, plasma MIF was increased by 2-fold whereas MIF content in the infarct myocardium was reduced by 34% compared to sham values (Figure 3A-B, both *P<0.05*). These reciprocal changes suggest that the early rise of plasma MIF level post MI is of cardiac origin. Moreover, at 72 h post-MI, plasma MIF remained higher than in sham mice, while MIF content in the infarct tissue was restored to a level higher than that observed in sham mice (Figure 3A-B).

### Leuocytes are an Important Source of Sustained Elevation of Circulating MIF following MI

**Figure 4:**
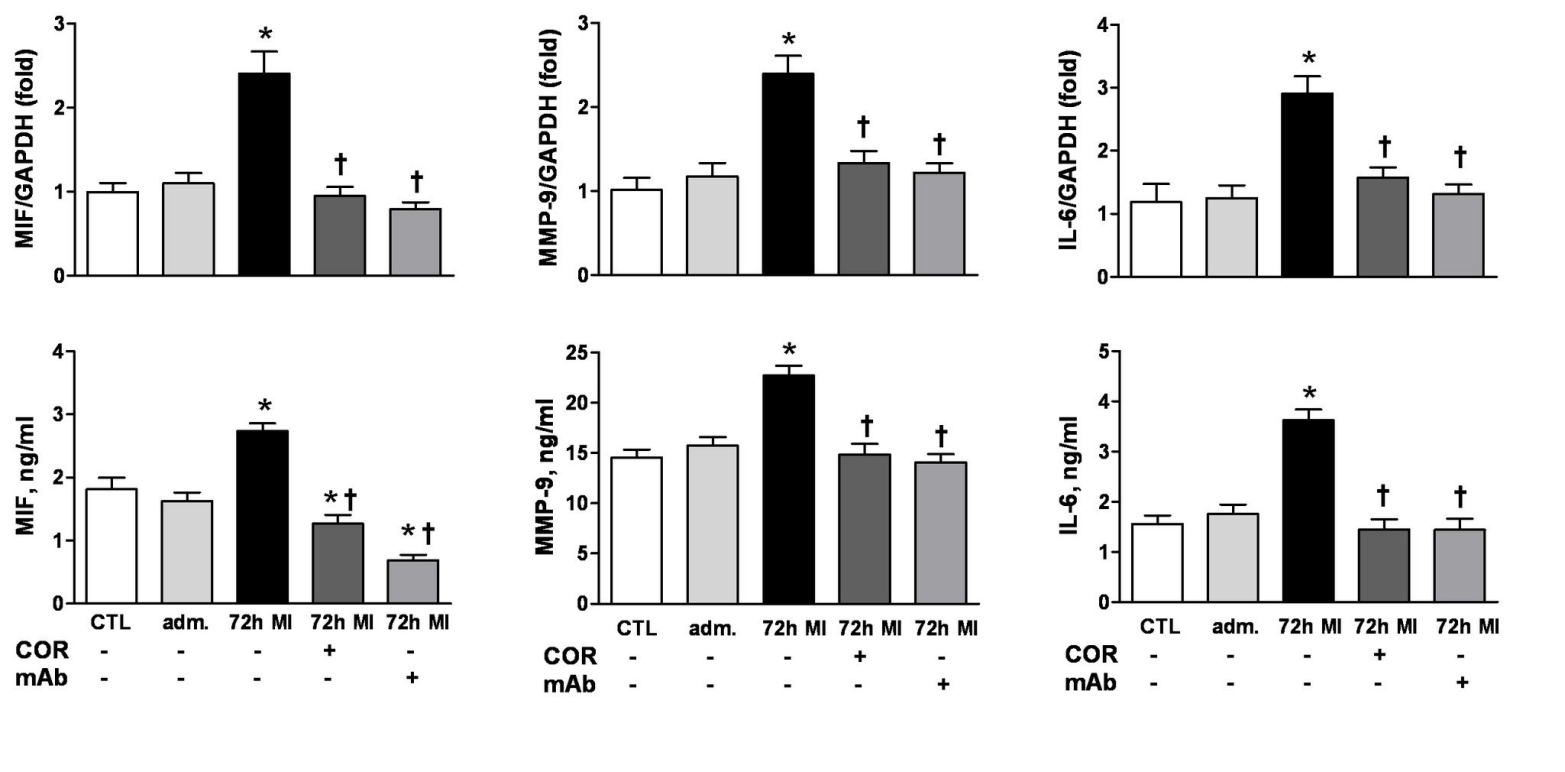
Inhibition of MIF attenuated MI-induced upregulation of MIF, MMP-9 and IL-6 by peripheral blood mononuclear cells (PBMCs). Increased mRNA (upper panels) and protein levels (bottom panels) of MIF, MMP-9 and IL-6 were observed in PBMCs from patients with MI at 72 h, while anti-MIF interventions either by MIF antagonist COR100140 (COR, 50 µM) or anti-MIF monoclonal antibody (mAb, 10 µg/ml) abolished upregulation of these pro-inflammatory mediators. n=10 for healthy controls (CTL). n=15 for admission (adm.) after MI and n=14 for 72 h MI groups. *P<0.05 vs. CTL, † *P*<0.05 vs. 72 h MI without intervention.

What is the significance of cardiac MIF release? In the setting of MI, activated peripheral blood leukocytes are recruited into the injured site, resulting in regional inflammation and wound healing, and these cells are another potential source of MIF. We therefore determined whether PBMCs from patients with MI were activated to produce MIF and inflammatory mediators *ex vivo* and whether inhibition of MIF could attenuate such a pro-inflammatory phenotype. In contrast to the rapid elevation in plasma MIF levels at admission after MI, significant increases in PBMC expression of MIF protein and mRNA compared to controls were observed at 72 h, but not at admission (Figure 3). Similar kinetics for MMP-9 and IL-6 were also observed (Figure 4). These results indicate a time-dependent activation of PBMC and mediators release after MI. Neutralisation of MIF with anti-MIF antibody or inhibition with a MIF antagonist, COR100140, *ex vivo* abolished the enhanced expression of MIF, MMP-9 and IL-6 mRNA and protein post-MI (Figure 4), suggesting that MIF mediates activation of PBMCs post-MI in an autocrine fashion. Further, in mouse infarct hearts, the density of macrophages was slightly higher at 3 h, but dramatically increased at 72 h post MI (Figure 3C-D). Collectively, the sustained rise of circulating MIF levels and restored MIF content in the infarct myocardium at 72 h are attributable to activation of circulating leukocytes and regional infiltration of leukocytes post-MI.

### MIF Alone or Synergistically with IL-1β Activates PBMCs

**Figure 5:**
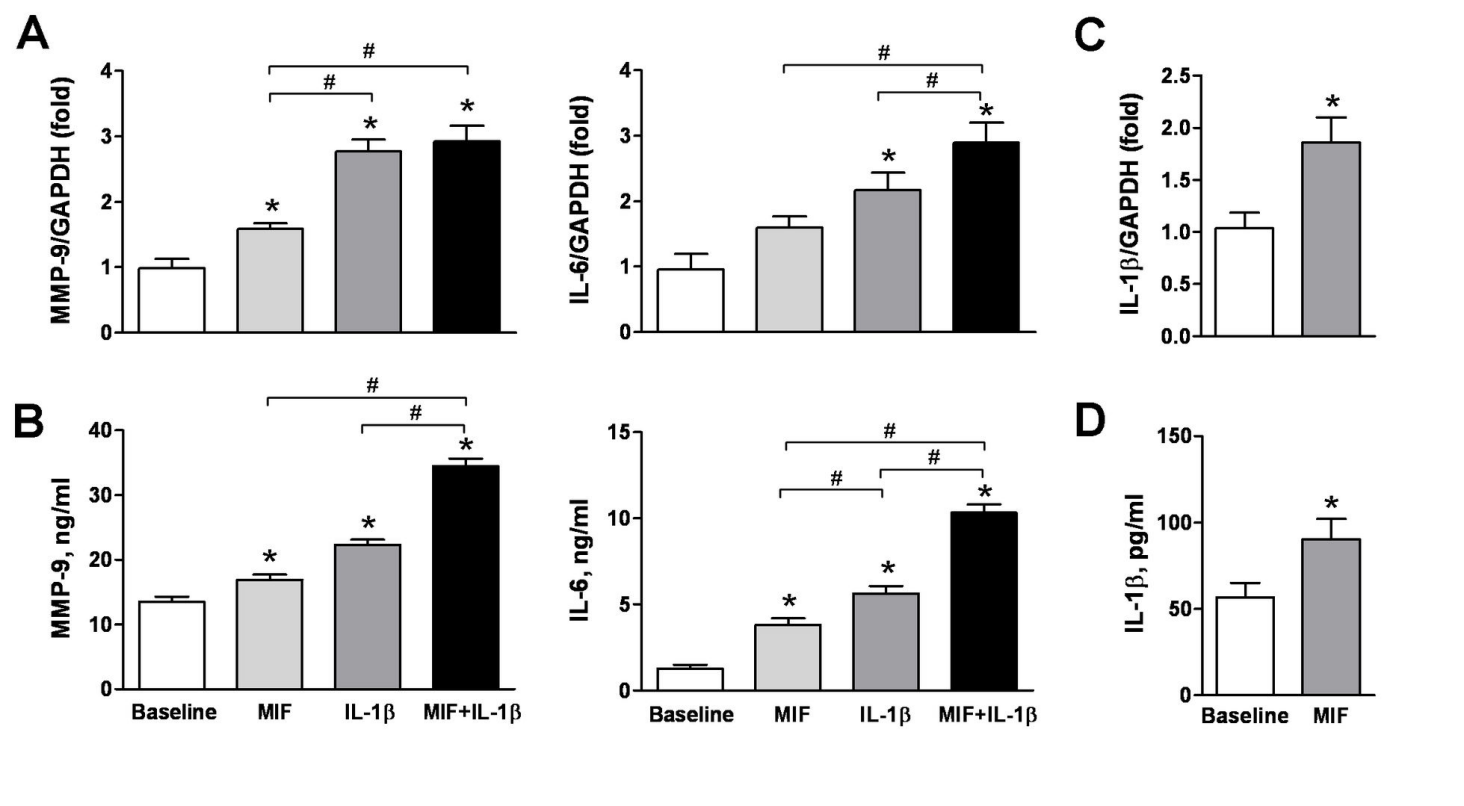
Exogenous MIF activates peripheral blood mononuclear cells (PBMCs) synergistically with IL-1β. Recombinant human MIF (rMIF, 5 ng/ml) and/or IL-1β (10 ng/ml) enhanced gene (**A**, upper panels) and protein (**B**, bottom panels) expression of MMP-9 and IL-6 in PBMCs from healthy controls (n=10). Upregulation of MMP-9 and IL-6 in PBMCs by combined treatment of rMIF and IL-1β was greater than that by single treatment. mRNA and protein levels of IL-1β of PBMCs stimulated by MIF was also significantly increased (**C**, **D**). *P<0.05 vs. baseline, ^#^ *P*<0.05.

**Figure 6:**
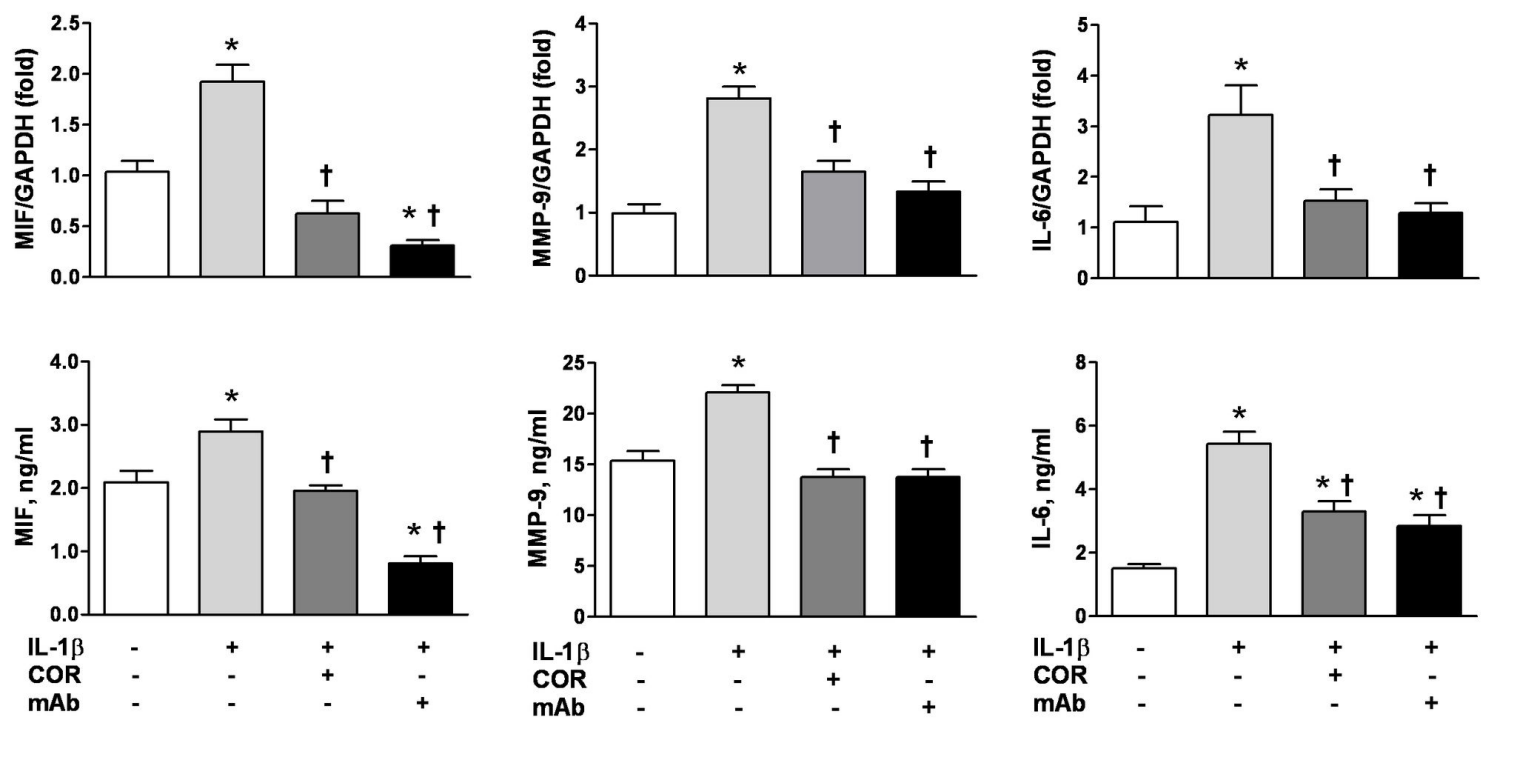
Anti-MIF interventions attenuated IL-1β induced activation of naïve peripheral blood mononuclear cells (PBMCs). IL-1β increased expression of MIF, MMP-9 and IL-6 at mRNA (upper panels) and protein levels (bottom panels) in PBMCs from healthy controls (n=10), while anti-MIF interventions by either MIF antagonist, COR100140 (COR, 50 µM), or anti-MIF monoclonal antibody (mAb, 10 µg/ml) attenuated these effects. *P<0.05 vs. baseline, † *P*<0.05 vs. IL-1β stimulation alone.

After observing time-dependent activation of PBMCs following MI, we next examined whether MIF was able to activate PBMCs directly. PBMCs from healthy volunteers were cultured with rMIF and/or IL-1β for 24 h. MIF or IL-1β alone each significantly upregulated PBMC MMP-9 and IL-6 mRNA and protein expression (Figure 5A-B). While the effect of IL-1β was more pronounced than that of MIF, treatment with both MIF and IL-1β increased expression of MMP-9 and IL-6 protein above that observed with either stimulus alone (Figure 5A-B). MIF also enhanced IL-1β mRNA and protein expression (Figure 5C-D). In another set of experiments on naïve PBMCs, increased expression of MIF, MMP-9 and IL-6 mRNA and protein induced by IL-1β stimulation was abolished by treatment with an anti-MIF monoclonal antibody or the MIF antagonist, COR100140 (Figure 6). These findings indicate that MIF directly induces MMP-9 and IL-6 expression by PBMCs, and also facilitates induction of these mediators by IL-1β.

### Anti-MIF Treatments Attenuate Inflammatory Cell Infiltration and Post-MI Cardiac Rupture

**Figure 7:**
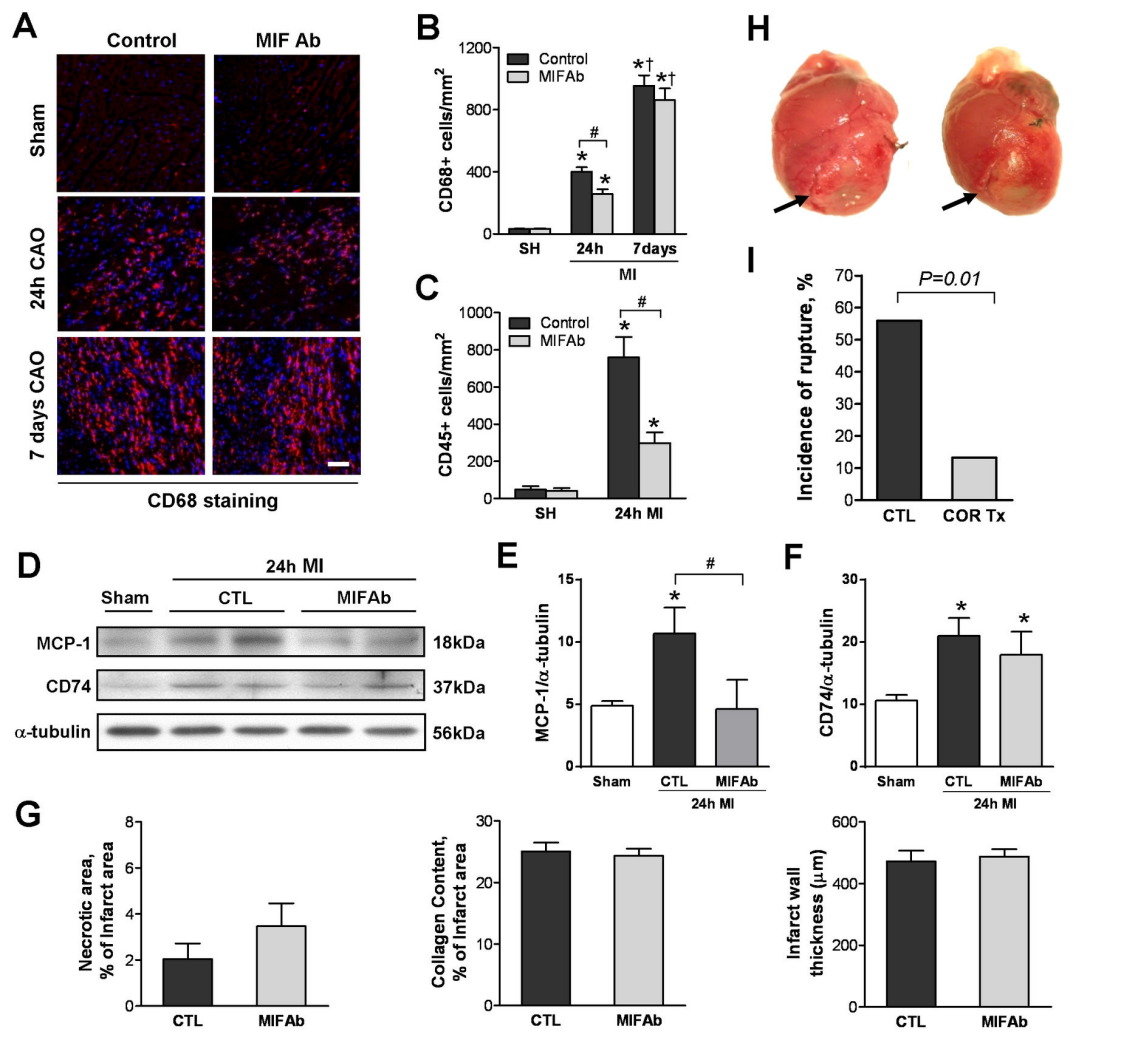
Influences of anti-MIF interventions in inflammatory cell infiltration, incidence of cardiac rupture and healing parameters in mice with MI. **A**. Representative images showing a temporal changes of macrophages (CD68+ cells, purple colour) by immunohistochemical staining in mice treated with isotype control IgG or anti-MIF polyclonal antibody (MIF Ab) post MI. Bar=100 μm. **B**, **C**. Grouped data showing that neutralizing MIF by anti-MIF Ab, i.p. given immediately after MI, significantly reduced density of macrophages (**B**, CD68 positive cells) and leukocytes (**C**, CD45+ cells) at 24 h, but had no effect on macrophage density at 7 days. n=3 for sham-operated groups (SH) and n=6-7 for each time point of MI groups. *P<0.*05* vs. respective SH, † *P<0.05* vs. respective 24 h MI, ^#^ *P*<0.05. **D**. Representative immunoblotting images for monocyte chemoattractant protein 1 (MCP-1) and CD74 in hearts from sham-operated or MI (24 h) mice treated with isotype control IgG or MIF Ab. **E**, **F**. Quantitative analysis of MCP-1 (**E**) and CD74 (**F**) expression. n=3/per sham group, n=5/per MI group. *P<0.*05* vs. sham, ^#^ *P<0.05*. **G**. Effects of anti-MIF Ab and isotype control IgG (CTL) treatment on healing parameters, i.e. size of residual necrotic myocardium and collagen content in the infarct area, and infarct wall thickness at 7 days post MI. n=4-6/group. **H**. Representative photos of ruptured hearts that occurred at 3-4 days after MI. Arrows indicate the rupture site around the border zone. **I**. Treatment with the MIF antagonist, COR100140, in the first 3 days post MI significantly reduced incidence of cardiac rupture. n=25 for untreated and 15 for treated groups.

To investigate the effect of anti-MIF intervention on inflammatory responses and cardiac remodeling following MI, we tested effects of different anti-MIF regimens in mice with MI. Immunohistochemical studies revealed that treatment with anti-MIF polyclonal antibody as a single dose given immediately after MI significantly reduced the density of both CD68+ macrophages and CD45+ leukocytes in the infarct region at 24 h, while macrophage infiltration at 7 days post MI was unaffected (Figure 7A-C). Immunoblotting revealed increased protein levels of CD74 and MCP-1 in the infarct myocardium at 24 h following MI compared to sham operated hearts. Treatment with anti-MIF antibody decreased MCP-1 expression but did no affect CD74 level at 24 h post-MI (Figure 7D-F). Healing parameters including size of residual necrotic area and collagen content in the infarct region, and infarct wall thickness, were not affected by anti-MIF treatment (Figure 7G). Further, mice treated with the MIF antagonist, COR100140, had markedly lower incidence of cardiac rupture within 7 days post-MI compared to controls (Figure 7H-I). Histological examination in mice dying of rupture showed a trend towards reduced infarct size in the anti-MIF treated group (37±5% vs. 33±4%, *P=0.067*). However, COR100140 treatment had no effect on LV dimension and FS determined by echocardiography at 4 weeks after MI, and final infarct size determined in all mice that survived to 4 weeks post-MI was similar between the two groups (Table 2).

**Table 2:** Echocardiographic data in mice with or without MIF antagonist after MI.

	Baseline		2 weeks MI		4 weeks MI	
	-COR	+COR	-COR	+COR	-COR	+COR
n	25	15	11	13	11	13
BW, g	30±1	28±2	28±4	26±5	30±1	29±1
HR, bpm	550±18	563±11	526±23	543±16	578±17	569±29
LVEDd, mm	3.88±0.10	3.70±0.07	5.50±0.1*	5.30±0.2*	5.67±0.27*	5.63±0.14*†
LVESd, mm	2.40±0.09	2.30±0.06	4.42±0.02*	4.25±0.17*	4.83±0.31*†	4.88±0.15*†
PWd th, mm	0.84±0.01	0.82±0.02	0.85±0.04	0.82±0.04	0.78±0.05	0.79±0.04
PWs th, mm	1.36±0.03	1.27±0.03	1.34±0.06	1.27±0.04	1.17±0.07*†	1.15±0.05*†
FS, %	36±3	38±1	20±3*	20±1*	15±2*†	13±1*†
IS, %	-	-	-	-	38.0±2.8	42.9±2.0

Values are expressed as mean±SEM. Animals received MIF antagonist, COR100140 (COR), treatment for first 3 days following myocardial infarction (MI). -COR, untreated control; +COR, treated group; BW, body weight; HR, heart rate; LVEDd, LEDSd, left ventricular end-diastolic or end-systolic dimension; PWd th, PWs th, posterior wall thickness at diastole or at systole; FS, fractional shortening; IS, infarct size. *P<0.05 vs. respective baseline. †*P*<0.05 vs. respective values at 2 weeks MI.

## Discussion

Our major focus in the present study is to document the potential influence of MIF in acute inflammation and its consequences following MI. In clinical and experimental studies, we documented dual cellular sources of MIF that may be responsible in sequence for the early and sustained elevation of circulating MIF post MI. We studied human PBMCs *ex vivo* and tested anti-MIF interventions in an *in vivo* mouse model. Our results demonstrate that expression of MIF was increased in PBMCs collected at 72 h, but not at 3 h after MI in humans; MIF resulted in activation of PBMCs *ex vivo* and facilitated activation by IL-1β; and MIF inhibition abolished PBMC activation associated with MI. *In vivo*, anti-MIF treatment suppressed inflammatory cell infiltration and reduced post-MI cardiac rupture. These findings support an important role of MIF in initiating and promoting inflammatory responses following MI.

Previous studies reported a rise of plasma MIF levels in patients around 4-6 h post MI [18,19]. We observed an early elevation of MIF in patients at admission, an average of 3 h after onset of symptoms. Such increase in plasma MIF levels was maintained up to 72 h post MI albeit there was a decline from the admission level. However, the cellular source of the plasma pool of MIF during the acute phase of MI is undefined. To address this, we matched *ex vivo* human cell culture experiments with determination of MIF plasma levels in patients at admission and 72 h following MI. Our results revealed a time discrepancy between rapid elevation of plasma MIF levels and delayed MIF expression by activated PBMCs *ex vivo*, suggesting that elevated plasma MIF after MI may be derived from different cellular sources. Cardiomyocytes and leukocytes, especially, monocytes and macrophages, are known to release MIF [11,30]. Unlike most cytokines, MIF is constitutively expressed and stored in intracellular pools, and can be readily released instantly without requiring *de novo* synthesis [11]. Previous experimental studies have shown that ischemia triggers cardiac MIF release into the coronary venous effluent and decreases cardiac MIF content [31,32]. Using a mouse MI model, we here observed a marked elevation in plasma MIF at 3 h post MI as well as a corresponding significant decrease in MIF content of the infarct myocardium. These reciprocal changes suggest that the early increase in circulating MIF after MI originates from the ischemic myocardium.

We attempted to define the role of MIF, known as a pro-inflammatory cytokine, in post-MI inflammation by studying the relationship of MIF with other inflammatory biomarkers. Interestingly, we observed a similar temporal changes of plasma MIF and IL-6 in MI patients, these changes were consistent with previous reports [33]. Moreover, we also observed significantly increased white blood cell and monocyte counts at admission. Although we do not have a direct evidence for the cellular source of IL-6, a previous study showed that in cultured cardiac fibroblasts, stimulation with β-adrenergic agonist, isoproterenol, evoked rapid release of IL-6 starting from 60 min [34]. Thus, upon acute myocardial ischemia and MI, cardiac cells are able to promptly release into circulation not only MIF, also other inflammatory molecules like IL-6. The close relationship between MIF and other inflammatory biomarkers suggests a possible role of MIF in avtivation of systemic inflammation.

Circulating monocytes are the precursor of local macrophages [35,36] and higher levels of white blood cells and monocytes at admission are associated with high mortality in patients with acute MI [5,6]. Thus, it is important to identify the mechanism by which PBMCs are activated. We next explored the potential that the elevated MIF activates PBMCs following MI in *ex vivo* studies. While the admission plasma MIF level was elevated, PBMCs obtained at this time point were not activated, evidenced by lack of difference in the expression of inflammatory mediators versus control PBMCs. PBMCs collected from the same subjects at 72 h exhibited enhanced expression of MIF, MMP-9 and IL-6. In mice, at 72 h, we observed restored MIF content in the infarct myocardium that contained robust infiltration of macrophages known to express MIF [11]. An early study reported in the infarct rat myocardium a co-localization of infiltrated macrophages and expression of MIF that peaked at day 3 post-MI [17]. These results indicate that inflammatory cells constitute an important cellular source for sustained elevation of circulating and cardiac MIF subsequent to the ultra-acute phase of MI.

Functionally, we confirmed important effects of MIF on leukocytes. Exogenous MIF directly activated naïve PBMCs evidenced by enhancing production of MMP-9 and IL-6, and facilitated the pro-inflammatory effect of IL-1β on these mediators. Moreover, inhibition of MIF *ex vivo* prevented activation of PBMCs after MI, and attenuated the effects of IL-1β stimulation on these mediators. Thus, MIF produced by PBMCs after MI acts in an autocrine fashion to upregulate other pro-inflammatory mediators. We and others have documented that increased MMP activity, especially MMP-9 primarily derived from inflammatory cells [37], plays a key role in cardiac inflammation, ECM degradation and adverse remodeling following MI [3,38,39]. Inhibition of MIF attenuated MMP-9 production from PBMCs post MI, suggesting that MIF is an important upstream activator of MMP-9 in this context. In clinical studies, elevated plasma MIF levels in patients with acute coronary syndrome also correlated with increased inflammatory markers such as C-reactive protein (CRP) and IL-6 [40]. MIF is known to induce expression of MCP-1 as well as monocyte-macrophage recruitment *in vivo* [25,41,42], indicating that cardiac release and accumulation of MIF also contributes to macrophage recruitment in the infarct region.

To further explore whether MIF expression and its pro-inflammatory actions following MI are detrimental, we applied anti-MIF interventions in the mouse MI model. Since macrophages in inflammatory tissues are differentiated from infiltrated monocytes after activation in circulation, their early presence (1-3 days) in the infarct myocardium represents inflammatory infiltrates, whilst the late presence (4-7 days) represents reparative macrophages [35,36]. We therefore studied effects of MIF neutralization on inflammatory cell infiltration at different time points by treatment of mice with a single dose of anti-MIF antibody given immediately after MI. Our results showed that MIF inhibition significantly reduced the density of leukocytes (CD45 positive cells) and macrophages (CD68 positive cells) at 24 h, but did not affect macrophage density at day-7 post-MI. Further, anti-MIF antibody treatment did not influence expression of MIF cell surface receptor, CD74, but significantly attenuated MCP-1 expression at 24 h after MI. These findings indirectly suggest that neutralizing MIF early after MI suppresses infiltration of inflammatory cells at the early phase at least partly through inhibition of MCP-1. Whereas such intervention potentially did not affect the recruitment of reparative macrophages at the later stage of MI. This conclusion is also supported by our histological analyses at 7 days post MI, showing no difference in clearance of infarct myocardium and collagen deposition between treated and control groups. However, since only one injection of anti-MIF antibody was given at the time of MI, it is possible that the antibody was no longer effective at the later stage, thus, may not affect reparative macrophages.

To test if MIF could be a therapeutic target to relieve acute and chronic cardiac remodelling, a small molecule MIF antagonist was administered for the first 3 days following MI. This intervention significantly reduced the incidence of post-infarct cardiac rupture. Taken together with our previous findings of suppressed inflammatory responses with smaller infarct size and preserved cardiac function following a severe ischemia-reperfusion (I/R) injury in MIF-deficient mice [25], and beneficial effects of anti-MIF therapy in other inflammatory disorders published by other groups [12,43]. These results identify MIF as a potential therapeutic target in inflammation post-MI, even though, we did not observe improvement in cardiac remodeling and function chronically in mice who received three days treatment with a MIF antagonist. This finding was in keeping with several previous reports showing that deletion of MMP-2 or p53 reduced risk of cardiac rupture without influencing chronic cardiac remodeling [44,45]. One likely explanation is that about 50% untreated mice that underwent severe cardiac remodeling died of rupture, leaving surviving mice with relative less myocardial injury and remodeling when compared to the treated group in which majority of mice survived to 4 weeks, this may have diminished the difference in the chronic cardiac remodeling between treated and untreated groups. This extrapolation is hinted at by a trend of relatively larger infarct size in untreated versus treated mice that died of cardiac rupture by day-7 after MI.

**Figure 8:**
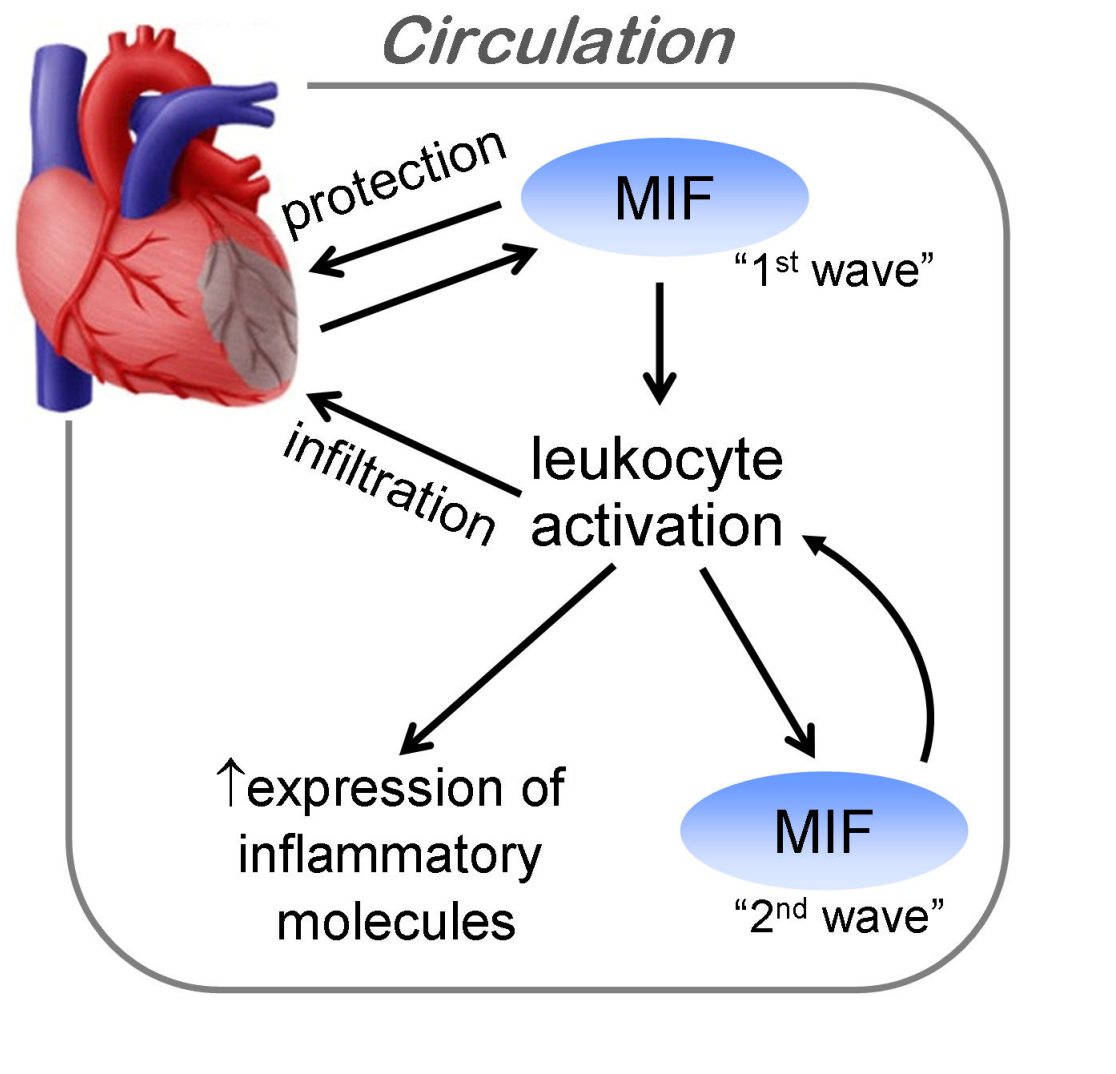
Schematic diagram depicting source of MIF and its action following MI. Early rise of MIF in circulation (first MIF wave) is released from the ischemic myocardium. Cardiac-derived MIF may exert cardioprotection in the setting of a brief ischemia, but under severe ischemic injury, activates circulating leukocytes resulting in increased expression of MIF (second MIF wave) and other inflammatory molecules such as MMP-9, IL-1β and IL-6. Activated inflammatory cells infiltrate into the infarct myocardium and enhance the regional inflammatory responses following MI.

Recent studies reported protective effects of MIF against cardiac I/R injury either by promoting glucose uptake via AMPK activation [31], suppressing oxidative stress [46] or inhibiting JNK-mediated apoptosis [32]. Notably, these beneficial effects of MIF are restricted to a brief ischemia of up to 20 min. When ischemia duration increases over 30 min (followed by reperfusion), MIF-mediated cardioprotection is no longer operative [25]. Koga et al also noted that when ischemia duration increased from 15 to 30 min, MIF mediated infarct size limitation, seen under 15 min ischemia, disappeared [46]. Instead, extent of inflammatory responses would dominant the final outcomes including infarct size, myocyte apoptosis and functional recovery. Under this scenario, deletion of MIF or MIF inhibitory intervention is beneficial by suppressing regional inflammation as showed by our previous [25] and the current study. Collectively, these findings support the notion that upon ischemic injury, MIF (1^st^ MIF wave, Figure 8) rapidly released from the myocardium, which may exert cardioprotection through these known mechanisms. However, with severe and prolonged ischemic injury, elevation of MIF then activates circulating PBMCs, which may induce local recruitment and sustain expression of MIF (2^nd^ MIF wave, Figure 8) and other inflammatory molecules, thereby enhancing the inflammatory responses post MI. It must be aware that inflammatory response is also necessary for healing process following elimination of dead cells and tissues by infiltrated macrophages, marked suppression of inflammatory response has been documented to be detrimental [47,48]. To what extent for inflammatory inhibition can deliver benefits to MI patients that is still a great challenge for contemporary medicine. However, with restrained therapeutic regimens (single dose of anti-MIF antibody or treatment of anti-MIF compound only in the first 3 days post-MI) targeting on ‘1^st^ MIF wave’ (Figure 8), we demonstrated reduced inflammatory infiltration at early stage without disturbing infarct healing and survival benefit. Our findings may cast light on the design of anti-inflammatory therapeutic regimens.

Several limitations merit consideration when interpreting the results of our study. Firstly, all animal experiments in this study were conducted in young mice, and significant differences may exist between young and aged animals. Therefore caution should be taken especially when a specific intervention is pursued on a therapeutic basis. Future studies on older animals would be helpful. Secondly, the sample sizes in the controls, stable angina and acute MI patients are relatively small precluding any definitive conclusions to be made. However, both our experimental and clinical studies provide novel insights into the pro-inflammatory action of MIF, which need to be validated in a larger scale study.

In conclusion, our findings on dynamic changes in MIF suggest dual cellular sources of the early and sustained rise in circulating MIF after MI. Elevated circulating MIF activates PBMCs to promote production of inflammatory mediators thereby enhancing inflammatory responses. Anti-MIF treatment attenuates post-MI inflammatory response and cardiac rupture, suggesting MIF as a potential therapeutic target in inflammation following MI.

## Supporting Information

Figure S1 **Dose-dependent inhibition of MMP-9 expression in peripheral blood mononuclear cells (PBMCs) by the MIF antagonist, COR100140 (COR).** **A**, PBMCs isolated from healthy human volunteers were stimulated with IL-1β (10 ng/ml) and/or treated with COR at different concentrations. Expression of MMP-9 in cultured media was determined by gelatin zymography and values ware normalized to the vehicle control and expressed as fold changes. **B**, representative gelatin SDS page showing an inhibitory effect of COR (50 μM) on MMP-9 expression. *P<0.05 vs. baseline value, ^#^ *P*<0.05. n=5-6 independent assays per group.
(TIF)
