# Correction: Pro-Inflammatory Action of MIF in Acute Myocardial Infarction via Activation of Peripheral Blood Mononuclear Cells

**DOI:** 10.1371/annotation/6e24b7bb-83c7-4887-9621-96b64acfb1c1

**Published:** 2013-11-13

**Authors:** David A. White, Lu Fang, William Chan, Eric F. Morand, Helen Kiriazis, Stephen J. Duffy, Andrew J. Taylor, Anthony M. Dart, Xiao-Jun Du, Xiao-Ming Gao

In the Funding Statement, the number of the grant from the National Health and Medical Research Council is incorrect. The correct grant number is: ID472600. 

