# Supplementary figures and images for: Pro-Inflammatory Action of MIF in Acute Myocardial Infarction via Activation of Peripheral Blood Mononuclear Cells
Source: PLoS One. 2013 Oct 1;8(10):e76206. doi: 10.1371/journal.pone.0076206 (PMC3788072; doi:10.1371/journal.pone.0076206)

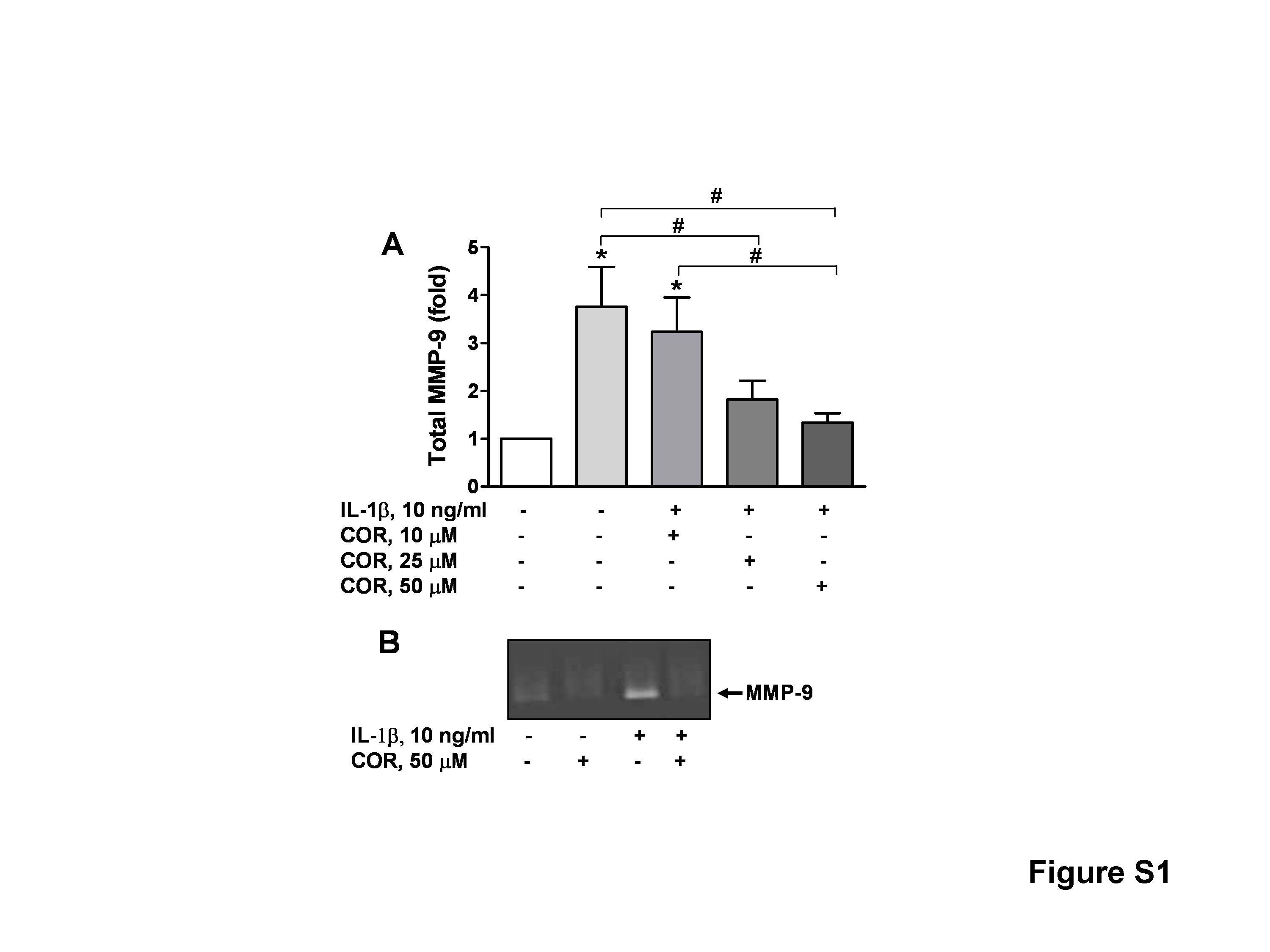

Supplement: Figure S1 — Dose-dependent inhibition of MMP-9 expression in peripheral blood mononuclear cells (PBMCs) by the MIF antagonist, COR100140 (COR). A, PBMCs isolated from healthy human volunteers were stimulated with IL-1β (10 ng/ml) and/or treated with COR at different concentrations. Expression of MMP-9 in cultured media was determined by gelatin zymography and values ware normalized to the vehicle control and expressed as fold changes. B, representative gelatin SDS page showing an inhibitory effect of COR (50 μM) on MMP-9 expression. *P<0.05 vs. baseline value, # P<0.05. n=5-6 independent assays per group. (TIF) [file pone.0076206.s001.tif]
